# Image-Level Data Augmentation for Radiomics-Based Classification of Vital Versus Non-Vital Persistent Cervical Lymph Nodes After Chemoradiotherapy in HNSCC

**DOI:** 10.3390/cancers18142293

**Published:** 2026-07-16

**Authors:** Sara Naccour, Assaad Moawad, Matthias Santer, Daniel Dejaco, Gerlig Widmann, Siegfried Kollotzek, Wolfgang Freysinger

**Affiliations:** 1 Department of Otorhinolaryngology-Head and Neck Surgery, Medical University of Innsbruck, 6020 Innsbruck, Austria; matthias.santer@tirol-kliniken.at (M.S.); daniel.dejaco@tirol-kliniken.at (D.D.); wolfgang.freysinger@i-med.ac.at (W.F.); 2 Datathings, 5, Rue de L’Industrie, L-1811 Luxembourg, Luxembourg; assaad.moawad@datathings.com; 3 University Hospital of Radiology, Medical University of Innsbruck, 6020 Innsbruck, Austria; gerlig.widmann@i-med.ac.at; 4 University Hospital of Radio-Oncology, Medical University of Innsbruck, 6020 Innsbruck, Austria; siegfried.kollotzek@i-med.ac.at

**Keywords:** radiomics, data augmentation, head and neck squamous cell carcinoma, feature selection, classification, chemoradiotherapy, machine learning

## Abstract

**Background:** Distinguishing vital from non-vital persistent cervical lymph nodes after chemoradiotherapy in HNSCC remains clinically challenging. We investigated whether image-level data augmentation improves CT-based radiomics classification for this task. **Methods:** We evaluated eight augmentation strategies and their 28 pairwise combinations in 55 patients, using Bayesian hyperparameter tuning with Optuna for parameter optimization. A radiomics pipeline comprising Radiomics features, five feature selectors, and seven classifiers was assessed using patient-level stratified 5-fold cross-validation. Configurations were ranked using a composite score defined as the mean of AUC, ACC and F1-score. **Results:** Feature selection improved the composite score from 0.659 to 0.742. The best augmented configuration, Window Contrast Variation, achieved a composite score of 0.803 and an AUC of 0.831, corresponding to an 8.2% relative point-estimate gain over feature selection alone and a 21.9% gain over the no-selection baseline when feature selection and augmentation were combined. **Conclusions:** These findings suggest that feature selection with optimized augmentation may enhance radiomics-based lymph node classification. However, individual augmentation-versus-baseline differences did not reach statistical significance in this limited sample, requiring confirmation in larger cohorts.

## Simple Summary

After chemoradiotherapy for HNSCC, cervical lymph nodes persist despite treatment, yet only a subset harbor active tumors. Identifying vital from non-vital nodes on CT scans helps guide decisions. We developed a machine learning pipeline that extracts image features from CT scans and tested whether augmenting training data with realistic transformations improves classification. This strategy improved classification performance by up to 21.9%, although differences did not reach statistical significance in this limited sample.

## 1. Introduction

Head and neck squamous cell carcinoma (HNSCC) accounts for approximately 4–5% of all cancers worldwide and remains a leading cause of cancer-related morbidity and mortality [1,2,3]. Primary concurrent chemoradiotherapy (CRT), alongside upfront surgery with adjuvant therapy, is among the standard treatment options for advanced-stage disease [4]. Following CRT, persistent cervical lymph nodes (pcLNs) are frequently observed on restaging CT; however, radiological persistency alone does not indicate residual viable tumor, as treatment-related inflammation, edema, and fibrosis can produce similar radiological appearances. Accurate classification of pcLNs as vital tumor-positive or non-vital (treatment-related changes only) is clinically essential, because vital nodes may require salvage neck dissection, whereas unnecessary surgery in non-vital cases increases patient morbidity without therapeutic benefit [5,6]. Reliably distinguishing between these two entities therefore remains a major clinical challenge.

Conventional post-CRT assessment relies on contrast-enhanced CT, which provides detailed anatomical information but has limited specificity for viable tumor when nodal morphology is confounded by post-treatment changes [6]. [^18^F]FDG PET/CT offers improved response assessment in many cases [7], but routine use is constrained by cost, availability, and false-positive uptake from post-radiation inflammation [5]. These limitations motivate the development of robust CT-based decision-support tools that can assess nodal viability without requiring additional specialized imaging.

Radiomics extracts quantitative features describing intensity, shape, and texture from medical images for use in machine learning classifiers [8,9]. This approach is well-suited to moderate-sized cohorts because features are explicitly defined, reproducible, and clinically interpretable. However, extracting many features from few patients creates a high overfitting risk, demanding rigorous feature selection and adherence to standardization efforts such as the Image Biomarker Standardization Initiative (IBSI) [10,11].

Despite the clinical relevance of post-CRT pcLN classification, few studies have addressed this specific task using radiomics. Dejaco et al. compared HR-contrast-CT and [^18^F]FDG-PET/CT for response evaluation of cervical lymph nodes after CRT, finding that neither modality alone achieved sufficient diagnostic accuracy [12]. In a related but distinct setting, Zhang et al. developed a radiomic model to predict lymph node response to induction chemotherapy, demonstrating the prognostic value of pre-treatment CT features [13]. A systematic review by Santer et al. surveyed applications of artificial intelligence to classify cervical lymph nodes in HNSCC, confirming the limited number of studies and the need for more robust approaches [14]. More recently, Santer et al. proposed the Neck-Persistency-Net, a three-dimensional convolutional neural network trained on the same patient cohort used in the present study, which achieved an AUC of 0.82 for distinguishing vital from non-vital pcLNs [15]. While deep learning models can achieve strong performance, they require greater computational resources and larger training datasets and offer less interpretability than radiomics-based approaches. Notably, no study has systematically investigated whether image-level data augmentation can improve radiomics-based classification of pcLN viability in HNSCC.

A common limitation across these studies is the small sample size typical of single-center medical imaging datasets. Data augmentation, which expands training data through label-preserving transformations, is a well-established strategy to address data scarcity and reduce overfitting [16,17,18]. Although augmentation and perturbation-based approaches have been explored in selected radiomics contexts, including breast imaging [19] and head-and-neck radiomics [20], their systematic, optimized evaluation within CT-based radiomics pipelines remains limited. Two domain-specific challenges account for this gap: first, radiomic features exhibit structured inter-feature correlations that augmentations must preserve, requiring image-level transformations before feature extraction [11]; second, CT augmentations must maintain the physical coherence of Hounsfield-unit values, restricting the admissible transformation space compared with natural-image domains.

In this study, we systematically investigated image-level data augmentation for radiomics-based classification of pcLN viability in HNSCC using contrast-enhanced CT. Building on our prior radiomics workflow [21], which includes preprocessing, feature extraction, feature selection, and leakage-controlled evaluation, we introduced augmentation as the primary experimental variable. We evaluated eight single-transform augmentation strategies and their 28 pairwise combinations under patient-level stratified 5-fold cross-validation. Our objectives were threefold: to quantify the individual and combined contributions of feature selection and image-level augmentation to classification performance; to determine which radiomic features are most discriminative for distinguishing vital from non-vital pcLNs; and to compare our radiomics approach with the deep learning benchmark of Santer et al. [15] on the same cohort. We hypothesized that systematic image-level augmentation, optimized according to three quality criteria (distribution preservation, robustness gain, and prediction consistency), would improve radiomics-based classification compared with unaugmented feature selection alone.

## 2. Materials and Methods

### 2.1. Patient Population

**Figure 1:**
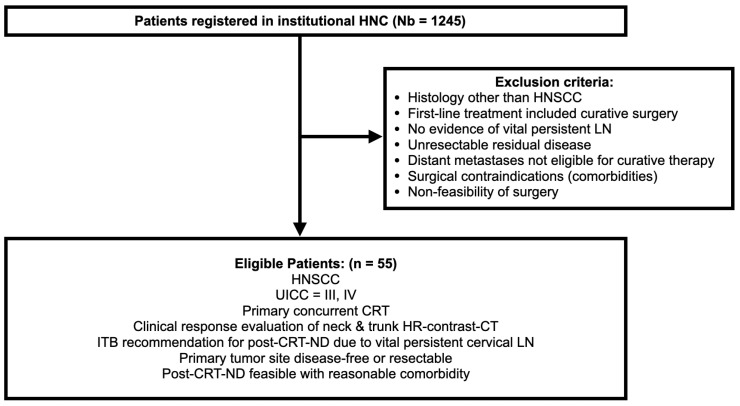
Patient selection flowchart. HNC, head and neck cancer; ITB, interdisciplinary tumor board; ND, neck dissection.

This retrospective study included patients from the institutional Head and Neck Cancer Registry of the Department of Otorhinolaryngology, Medical University of Innsbruck (Austria), documented between January 2008 and December 2023. The cohort comprised individuals with incident, histopathologically confirmed advanced HNSCC (Union for International Cancer Control [UICC] stage III or IV, including carcinoma of unknown primary) who received primary concurrent chemoradiotherapy. Eligible patients were those with clinically or radiologically persistent cervical nodal disease following chemoradiotherapy who subsequently underwent salvage or restaging neck dissection based on interdisciplinary tumor-board decision-making. Patients were excluded if they had non-HNSCC histology, had received first-line curative surgery, or were not eligible for curative therapy. Following application of these criteria, the final analytic cohort comprised 55 patients: 27 classified as vital/tumor-positive and 28 as non-vital/tumor-negative, based on histopathological assessment of the neck-dissection specimens. A detailed overview of the selection workflow is provided in Figure 1.

Among the 55 patients who met the inclusion criteria, 47 (85.5%) were men and 8 (14.5%) were women. At initial diagnosis, 10 patients (18.2%) were 50 years of age or younger, 18 (32.7%) were 51 to 60 years of age, 19 (34.5%) were 61 to 70 years of age, and 8 (14.5%) were 71 years of age or older. The oropharynx was the most common primary tumor site, observed in 26 patients (47.3%), followed by carcinoma of unknown primary in 9 (16.4%), hypopharynx in 8 (14.5%), oral cavity in 7 (12.7%), larynx in 3 (5.5%), and both nasopharynx and nasal cavity or paranasal sinuses in 1 patient each (1.8%). Among the 35 patients with oropharyngeal carcinoma or carcinoma of unknown primary, p16 status was positive in 13 (37.1%) and negative in 22 (62.9%). Clinical T stage at diagnosis was cT0 in 9 patients (16.4%), cT1 in 7 (12.7%), cT2 in 13 (23.6%), cT3 in 11 (20.0%), and cT4 in 15 (27.3%). After primary concurrent chemoradiotherapy followed by neck dissection, histopathological analysis showed viable cervical lymph nodes in 27 patients (49.1%), whereas 28 (50.9%) had a complete nodal response.

Staging followed the UICC 8th Edition [22]. In accordance with National Comprehensive Cancer Network (NCCN) guidelines [4], the interdisciplinary tumor board recommended primary concurrent chemoradiotherapy. Chemotherapy consisted of either cisplatin or mitomycin-C plus 5-fluorouracil, administered in two cycles. Radiotherapy delivered 70 Gy in 35 fractions over 7 weeks to the primary tumor and pathological lymph nodes; high-risk involved nodal levels received 60 Gy and low-risk elective levels received 54 Gy, both in 30 fractions. Treatment response was assessed 8–10 weeks after CRT completion using contrast-enhanced CT of the neck and thorax, combined with endoscopic examination and directed biopsies from the primary tumor site when indicated [23]. Response was classified according to World Health Organization criteria [24,25]. Complete staging and treatment methodology has been described previously [15].

Because radiomic features are sensitive to image acquisition, an important question is whether any apparent class signal could instead reflect differences in scanner, reconstruction, or target-node size between the vital and non-vital groups rather than tumor viability. The detailed clinical and demographic characteristics of the broader cohort and imaging protocol have been reported previously [15] and are summarized above; here we focus on the acquisition- and size-related factors most relevant to radiomic feature stability, stratified by histopathological nodal status in Table 1. These values were extracted from the current analytic cohort for the present analysis. Age, sex, scanner vendor, reconstruction slice thickness, and acquisition year were obtained from the restaging CT DICOM metadata (patient birth date and study date for age and acquisition year; manufacturer and slice-thickness tags for the acquisition characteristics), whereas the nodal-size descriptors (maximum three-dimensional diameter and minor-axis length, the latter used as a short-axis proxy) were derived from the segmented target lymph node using PyRadiomics shape features. Because nodal-size distributions contained occasional segmentation-related outliers, size descriptors are reported as medians with interquartile ranges, and group comparisons are presented descriptively, without formal significance testing, given the limited cohort size. No obvious descriptive imbalance between the two groups was apparent for the characteristics that could be stratified: age, sex, scanner vendor, and slice thickness were comparable, and median nodal size differed only modestly. Primary tumor site, p16 status, clinical T stage, and detailed treatment-regimen variables were documented for the broader cohort, but could not be reliably stratified by histopathological target-node status in the present analytic dataset. These variables were therefore reported only descriptively at the cohort level and were not included in the nodal-status-stratified baseline table. However, all patients followed the same core treatment pathway, with primary concurrent chemoradiotherapy followed by restaging at the protocol-defined interval.

### 2.2. Contrast-Enhanced CT Scans and Segmentation

Contrast-enhanced CT examinations were obtained at the Department of Radiology, Medical University of Innsbruck, using the institutional head and neck imaging protocol, with reconstruction on a 512×512 matrix and a nominal protocol comprising a 2 mm slice thickness, 24 × 1.2 mm collimation, and a pitch of 0.45 [15]. Because the restaging examinations were collected retrospectively over an extended period (2008–2021), the analyzed scans were acquired on several GE and Siemens multidetector CT systems (predominantly GE Discovery CT750 HD^®^ and LightSpeed VCT^®^, GE-Medical Systems, Vienna, Austria; and Siemens SOMATOM^®^ and Sensation^®^ systems, Siemens Healthineers, Erlangen, Germany), with reconstruction slice thicknesses ranging from 0.625 to 3.75 mm (median 2.5 mm). The distribution of scanner vendor and slice thickness by nodal status is summarized in Table 1. The scan range extended from the frontal sinus to the upper mediastinum, and coronal and sagittal reformations were generated from the axial dataset. An iodinated contrast agent (Jopamiro 370^®^, Bracco Austria GmbH, Vienna, Austria) was administered intravenously according to institutional protocols. All examinations were archived and exported in Digital Imaging and Communications in Medicine (DICOM) format via DeepUnity Diagnost (Dedalus, DH Healthcare GmbH, Bonn, Germany) and the institutional picture archiving and communication system (PACS^®^, Cerner Corporation, North Kansas City, MO, USA). CT scans were interpreted by board-certified head and neck radiologists and radiation oncologists and reviewed at the interdisciplinary tumour board. Cervical lymph nodes with residual post-treatment findings after primary chemoradiotherapy were classified as persistent in analogy to the Response Evaluation Criteria in Solid Tumours version 1.1 (RECIST 1.1) [26]. Additional details on staging and treatment are reported in [15].

For subsequent volumetric analysis, each post-treatment restaging CT was imported into the Elements^®^ platform (BrainLab, Munich, Germany). One cervical lymph node per patient was selected by the interdisciplinary tumor board as the target node, defined as the node with confirmed or suspected residual viable disease; if multiple suspicious nodes were present, the node of greatest clinical concern was selected. For the analyzed cohort, nodal status was determined by histopathological assessment of post-chemoradiotherapy neck dissection specimens. The full cohort and outcome-labeling methodology have been described previously [15]. The target node was manually segmented slice-by-slice in the axial, coronal, and sagittal planes to define a three-dimensional region of interest (ROI). Segmentations were quality-checked by senior head and neck radiologists with more than 15 years of subspecialty experience. The final ROIs were exported in DICOMDIR format for downstream radiomics feature extraction and analysis.

### 2.3. Data Preprocessing Pipeline

To ensure geometric and intensity consistency for quantitative analysis, DICOM CT scans retrieved from the clinical PACS were converted to Nearly Raw Raster Data (NRRD) format using a processing pipeline implemented in Python (v3.10.X) with pydicom (v2.4.0), SimpleITK (v2.2.0), and pynrrd (v1.0.0). De-identification was achieved by selective extraction. Only pixel data and the spatial metadata required for volumetric reconstruction (image position, orientation, and voxel spacing) were transferred to the output NRRD files. Thus, protected health information present in the original DICOM headers was inherently excluded, requiring no explicit tag scrubbing. This procedure aligned with established best practices for preparing de-identified medical imaging datasets for research use [27,28], while preserving full spatial metadata and orientation descriptors in a rigorously defined file structure [29]. Each converted volume subsequently underwent automated integrity verification to confirm consistent inter-slice spacing, the expected left–posterior–superior (LPS) anatomical orientation, non-zero volume dimensions along all three axes, and matching geometry between the CT volume and its corresponding segmentation mask. These checks mitigated well-characterized sources of variability in clinical DICOM data; all verified volumes were stored in LPS orientation.

Using the manually curated lymph-node masks, each verified volume was cropped to the axis-aligned minimum bounding box of all non-zero mask voxels, extended by a 5 mm margin to retain peri-nodal context. Cropped volumes were resampled to isotropic $1\times 1\times 1\;{\mathrm{mm}}^{3}$ resolution (B-spline interpolation for CT, nearest-neighbor for masks) [11,30]. Resampling preceded denoising to avoid anisotropic smoothing [31]. A recursive Gaussian filter (σ=0.5mm; SimpleITK IIR approximation [32]) was applied to attenuate acquisition noise. Intensities were clipped to ([−1024,3071]HU) and standardized via per-volume Z-score normalization, which subtracts the mean and divides by the standard deviation of each volume independently. Statistics were computed exclusively over foreground voxels (HU>−900) to exclude air, which otherwise dominates the volume and skews the normalization towards clinically irrelevant background. Because the transform is computed per volume, it introduces no cross-partition leakage [33]. For radiomics analysis, however, feature extraction was performed on the HU-preserved representation before per-volume Z-score normalization, as detailed in Section 2.5. The Z-score-normalized volumes were symmetrically padded to (128×128×128) voxels, the smallest power-of-two cube enclosing all cropped volumes and matching the input dimensions of the Neck-Persistency-Net [15] for direct comparison, using the per-volume minimum intensity for CT and zero for masks.

No inter-subject registration was performed, as all examinations were acquired in a consistent head-and-neck orientation. No organ-specific intensity windowing was applied during preprocessing, as collapsing the native HU dynamic range would discard attenuation differences required for texture and first-order feature computation. All preprocessing steps were executed in the order described above.

### 2.4. Data Augmentation

A fundamental challenge in radiomics is that high-dimensional feature spaces can substantially exceed the available sample size, increasing the risk of overfitting [10,34]. In the present study, 107 radiomic features are extracted from 55 patients (p>n), creating a severely underdetermined system in which classifiers can fit noise rather than generalizable signal. Moreover, probabilistic classifiers yield more reliable parameter estimates and better-calibrated decision boundaries as the effective training set grows, because larger samples reduce the variance of estimated class-conditional distributions and improve approximation of the true data-generating process [35,36]. Data augmentation directly addresses both limitations by expanding training data through clinically plausible, label-preserving transformations that simultaneously increase the effective sample size and improve robustness to acquisition-related nuisance variation [16,17,18,37].

Critically, radiomic descriptors exhibit structured inter-feature dependencies arising from shared mathematical formulations (e.g., grey-level co-occurrence and run-length matrices) [10,11]. Feature-space oversampling techniques such as SMOTE [38] can disrupt these correlations, producing synthetic samples that may not correspond to physically plausible anatomy. We therefore adopted image-level augmentation as the primary strategy.

#### 2.4.1. Initial Parameter Bounds and Search-Space Design

Initial parameter bounds for each augmentation strategy were informed by literature-reported augmentation ranges in medical imaging and expected magnitudes of clinically plausible variability in head-and-neck CT [16,17,39]. These bounds were validated by visual inspection of representative augmented volumes from a held-out subset of the training data to avoid non-physical artifacts. Each parameter was discretized into five levels spanning a conservative-to-aggressive spectrum. The parameter ranges reported in the following subsections are the final values selected by the optimization procedure (Section 2.4.13), not the initial search bounds.

#### 2.4.2. Augmentation Strategies and Leakage Control

We evaluated 36 augmentation configurations: eight single-transform strategies and all 82=28 pairwise combinations. To prevent data leakage, patient-level partitioning was carried out before any augmentation, and augmented samples were generated only within the training partition of each fold [40]. All transformations were applied to the preprocessed volumes described in Section 2.3. Intensity parameters reported below are expressed in Hounsfield units (HU) on the pre-normalization scale.

Throughout this section, $N(\mu ,\sigma^{2})$ denotes the Gaussian distribution, U(a,b) the continuous uniform distribution on [a,b], clip(x,a,b)=min(max(x,a),b), and ∗ spatial convolution.

#### 2.4.3. Spatial Augmentation Strategies

Five spatial augmentation strategies were implemented to emulate positioning variability, anatomical differences, and non-rigid deformation [16,17,37,39]. For all spatial transforms, CT volumes used B-spline interpolation (trilinear for elastic deformation), and masks used nearest-neighbor interpolation; out-of-domain voxels were filled with the per-volume minimum intensity for CT and zero for masks.

#### 2.4.4. Geometric Transformations

Geometric augmentation was used to emulate patient-positioning variability during CT acquisition. Let $x={\left(x,y,z\right)}^{T}$ denote a voxel coordinate; the transformed coordinate $x^{\prime }$ is defined as

(1) $$x^{\prime }=s\;Rx+t,$$

where R is a 3D rotation matrix with angles $\theta_{x},\theta_{y},\theta_{z}\in \left[-8{}^{\circ},+8{}^{\circ}\right]$ applied in the fixed composition order z→y→x, s∈[0.9,1.05] is an isotropic scaling factor, and $t={\left(t_{x},t_{y},t_{z}\right)}^{T}$ is a translation vector with $t_{i}∼U\left(-0.03\;L_{i},\;+0.03\;L_{i}\right)$, where $L_{i}$ is the extent of the volume along axis *i*.

#### 2.4.5. Elastic Deformation

Elastic deformation was used to introduce smooth, non-rigid distortions that emulate plausible anatomical variability [37,39]. A displacement field $u\left(x\right)={\left(u_{x},u_{y},u_{z}\right)}^{T}$ was generated by smoothing random noise and scaling its amplitude:

(2) $$u\left(x\right)=\alpha \cdot \left(G_{\sigma }*n\right)\left(x\right),$$

where $n\left(x\right)∼U{\left(-1,+1\right)}^{3}$, $G_{\sigma }$ is a 3D Gaussian kernel with σ∈[11.4,15.9] mm controlling spatial smoothness, and α∈[21.0,63.6] mm controls displacement amplitude. Both α and σ were converted to voxel units using the image spacing; the kernel was truncated at 4σ. The deformed image was obtained by

(3) $$I^{\prime }\left(x\right)=I\;\left(x+u(x)\right),$$

with displaced coordinates clipped to the image domain.

#### 2.4.6. Shearing

Shearing was used to emulate small positioning deviations relative to the scanner coordinate axes [16,17]. A 3D affine shear about the volume center was applied as

(4) $$x^{\prime }=Hx,\;H=\left(\begin{array}{ccc} 1 & \tan\phi_{xy} & \tan\phi_{xz}\\ 0 & 1 & \tan\phi_{yz}\\ 0 & 0 & 1 \end{array}\right),$$

where $\phi_{xy}\in \left[-9.1{}^{\circ},+9.1{}^{\circ}\right]$, $\phi_{xz}\in \left[-6.6{}^{\circ},+6.6{}^{\circ}\right]$, and $\phi_{yz}\in \left[-2.2{}^{\circ},+2.2{}^{\circ}\right]$. The larger in-plane range reflects the dominant mode of patient-positioning variability; the upper-triangular parameterization avoids redundancy with rotation.

#### 2.4.7. Anisotropic Scaling

Anisotropic scaling was used to introduce axis-specific size variation reflecting inter-patient anatomical differences and acquisition-related anisotropy, where in-plane and through-plane voxel spacings may differ [16,17,18]. The transformation of the volume center was defined as

(5) $$x^{\prime }=S_{\mathrm{aniso}}\;x,\;S_{\mathrm{aniso}}=\left(\begin{array}{ccc} s_{x} & 0 & 0\\ 0 & s_{y} & 0\\ 0 & 0 & s_{z} \end{array}\right),$$

where $s_{x},s_{y}\in \left[0.94,1.09\right]$ and $s_{z}\in \left[0.94,1.12\right]$. A wider range was permitted along the through-plane axis to account for greater variability in slice spacing.

#### 2.4.8. Translation with Shearing

To emulate co-occurring translational and angular setup deviations [16,17], a composite affine transformation combining translation and in-plane shearing was applied:

(6) $$x^{\prime }=H_{\mathrm{ip}}\;x+t,$$

where $t={\left(t_{x},t_{y},t_{z}\right)}^{T}$ is a translation vector with $t_{x,y,z}\in \left[-7.6,+7.6\right]$ mm and

(7) $$H_{\mathrm{ip}}=\left(\begin{array}{ccc} 1 & \tan\phi_{xy} & 0\\ 0 & 1 & 0\\ 0 & 0 & 1 \end{array}\right),$$

with $\phi_{xy}\in \left[-8.2{}^{\circ},+8.2{}^{\circ}\right]$. Only the in-plane shear component was retained to model the dominant mode of combined translational and angular setup error.

#### 2.4.9. Intensity Modifications

Intensity-based augmentation was used to emulate scanner- and protocol-dependent variability in CT acquisition and reconstruction [16,17]. Let I(x) denote the CT intensity at voxel position x and $I^{\prime }\left(x\right)$ the corresponding augmented intensity. For each augmented instance, one to three of the following four operations were randomly selected without replacement and applied sequentially:

(i) Additive Gaussian noise:
  $$I^{\prime }\left(x\right)=I\left(x\right)+\epsilon ,\;\epsilon ∼N\left(0,\sigma^{2}\right),\;\sigma \in \left[5,8\right]\;\mathrm{HU}.$$
(ii) Global intensity bias:
  $$I^{\prime }\left(x\right)=I\left(x\right)+b,\;b∼U\left(-40,+40\right)\;\mathrm{HU},$$
  where the range spans typical inter-scanner calibration offsets [16,17].
(iii) Gaussian blur:
  $$I^{\prime }\left(x\right)=\left(I*G_{\sigma_{b}}\right)\left(x\right),\;\sigma_{b}\in \left[0.6,0.8\right]\;\mathrm{voxels},$$
  where $G_{\sigma_{b}}$ is a 3D Gaussian kernel with standard deviation $\sigma_{b}$ in voxel units, truncated at $4\sigma_{b}$.
(iv) Multiplicative contrast scaling:
  $$I^{\prime }\left(x\right)=\left(I\left(x\right)-\bar{I}\right)\;c+\bar{I},$$
  where $\bar{I}$ is the global mean intensity of the volume and c∼U(0.85,1.15), introducing mild linear Contrast Variation within the range of protocol-dependent reconstruction differences [17].

#### 2.4.10. CT Window Variation

CT window variation was used to emulate local intensity variability arising from scanner-, reconstruction-, and protocol-dependent differences in head-and-neck CT. The transformation is parameterized by an intensity window, defined by a center and width in the Hounsfield-unit domain, but it should not be interpreted as a clinical display window/level adjustment. In clinical CT, window and level settings affect only image display and do not modify the stored HU values. In contrast, the present augmentation applies a controlled synthetic intensity perturbation within a sampled HU interval. Starting from a standard soft-tissue baseline window (center $c_{0}=40$ HU, width $w_{0}=400$ HU), augmented parameters were sampled as

(8) $$\begin{array}{cc} c^{\prime } & =c_{0}+\Delta c,\;\Delta c∼U\left(-12.2,+12.2\right)\;\mathrm{HU},\\ w^{\prime } & =\max\;\left(100,\;w_{0}\cdot s_{w}\right),\;s_{w}∼U\left(0.79,1.22\right), \end{array}$$

where the width is clamped to a minimum of 100 HU to prevent implausibly narrow intensity intervals. The resulting HU interval $\left[c^{\prime }-w^{\prime }/2,\;c^{\prime }+w^{\prime }/2\right]$ was used for linear intensity normalization followed by clipping,

(9) $$\tilde{I}\left(x\right)=\mathrm{clip}\;\left(\frac{I\left(x\right)-\left(c^{\prime }-w^{\prime }/2\right)}{w^{\prime }},\;0,\;1\right),$$

and a mild monotonic tone-curve perturbation was applied via gamma correction with γ∼U(0.95,1.05). The perturbation was applied selectively: voxels within the sampled HU interval underwent normalization (Equation (9)), gamma correction, and inverse mapping back to the Hounsfield-unit scale, whereas voxels outside the interval retained their original intensities. Formally,

(10) $$I^{\prime }\left(x\right)=\left\{\begin{array}{cc} {\left(\tilde{I}\left(x\right)\right)}^{\gamma }\;\left(w^{\prime }\right)+\left(c^{\prime }-w^{\prime }/2\right), & \mathrm{if}\;c^{\prime }-w^{\prime }/2\leq I\left(x\right)\leq c^{\prime }+w^{\prime }/2,\\ I(x), & \mathrm{otherwise}, \end{array}\right.$$

where the first branch applies the normalization, gamma correction, and inverse mapping back to Hounsfield units only within the sampled interval, and the second branch preserves original intensities. The selective formulation was adopted because radiomic features, including first-order statistics and texture descriptors, depend on HU-based attenuation differences within the segmented region. Voxels inside the sampled interval undergo a bounded monotonic intensity perturbation and are mapped back to the HU scale, while voxels outside the interval, such as air, bone, and high-contrast structures, remain unchanged. The augmented volumes therefore remain compatible with standardized radiomics feature extraction.

#### 2.4.11. Contrast Variability

To emulate variability in contrast enhancement across acquisitions [17,18,34], we applied selective intensity scaling that modulates enhancing structures while preserving background tissue. A binary indicator mask was constructed by thresholding at the τ-th intensity percentile:

(11) $$1_{\tau }\left(x\right)=\left\{\begin{array}{cc} 1, & I\left(x\right)>P_{\tau },\\ 0, & \mathrm{otherwise}, \end{array}\right.$$

where $P_{\tau }$ denotes the τ-th percentile of voxel intensities in the volume (τ=66, selected by Bayesian optimization with Optuna). A spatially smooth blending mask was obtained by convolving the indicator with a Gaussian kernel:

(12) $$m\left(x\right)=\left(G_{\sigma_{m}}*1_{\tau }\right)\left(x\right),\;\sigma_{m}=1.5\;\mathrm{voxels},$$

yielding m(x)∈[0,1] with gradual transitions at region boundaries; the kernel was truncated at $4\sigma_{m}$. An enhanced volume was computed by scaling only the above-threshold intensity component:

(13) $$I_{\mathrm{enh}}\left(x\right)=P_{\tau }+\left(I\left(x\right)-P_{\tau }\right)\;s_{e},\;s_{e}∼U\left(0.94,1.17\right).$$

The final augmented intensity was obtained by blending the original and enhanced volumes:

(14) $$I^{\prime }\left(x\right)=\left(1-m(x)\right)\;I\left(x\right)+m\left(x\right)\;I_{\mathrm{enh}}\left(x\right).$$

**Figure 2:**
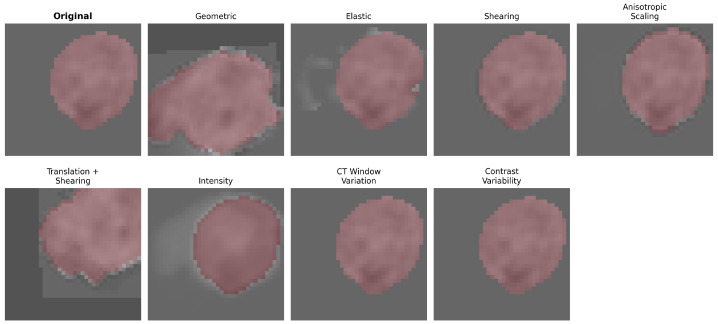
Representative axial slices of a vital (histopathologically confirmed) persistent cervical lymph node from a post-CRT restaging CT of an HNSCC patient (institutional cohort, Medical University of Innsbruck). The segmentation mask is shown in red. The original CT image (top left) is shown alongside the results of the eight augmentation strategies.

Figure 2 shows representative axial slices before and after each of the eight augmentation strategies. The parameter ranges reported above and the augmentation factor for each strategy were selected through a two-stage process of grid search and Optuna hyperparameter optimization, followed by Pareto-front selection across three quality criteria (Section 2.4.13).

#### 2.4.12. Combination Strategies

All twenty-eight pairwise combinations of the eight base strategies were evaluated. Operations were applied sequentially, with spatial transforms preceding intensity modifications to avoid interpolating already-modified voxel values. Individually optimized parameters were reused without joint re-optimization, reducing the combinatorial search space while leveraging per-strategy tuning.

#### 2.4.13. Hyperparameter and Augmentation Factor Optimization

Transformation parameters and the augmentation factor *f* (the ratio of augmented to original samples) were optimized jointly in a two-stage framework. To keep computational cost manageable, all candidate configurations were first evaluated on volumes downsampled to $64^{3}$ voxels. The search comprised more than 500 trials per strategy across both stages. This downsampling reduced computation time per volume by approximately two orders of magnitude compared with the native resolution, while preserving enough spatial detail for the three quality criteria, namely distribution preservation, robustness gain, and prediction consistency, to remain informative.

Within each strategy’s search, each trial evaluated a candidate configuration on a randomly drawn, class-balanced 20-patient subset (ten patients per class, approximately one-third of the cohort), with the random seed fixed for reproducibility. Different random subsets were used across strategies, reducing the dependence of parameter selection on a single arbitrary partition of the data. This subset size represented a pragmatic compromise: it was small enough to allow extensive parameter exploration, yet large enough for the quality criteria to provide informative relative rankings. These criteria were used solely to guide augmentation parameter selection and were deliberately decoupled from the final classification metrics. The 20-patient subsets used for this preliminary augmentation parameter optimization were drawn from the full cohort and were not excluded from the subsequent cross-validation folds. Therefore, they were not independent test cases. However, the augmentation parameters were chosen using predefined quality criteria, not the final cross-validated classification metrics.

We deliberately chose not to nest this tuning procedure inside the cross-validation loop used for final model evaluation. The tuning step was intended to rank augmentation settings by relative data quality, not to estimate generalization performance. Nesting the search within the cross-validation would have blurred the boundary between parameter tuning and model evaluation, introducing a risk of optimistic bias (Section 2.6). We acknowledge that, because each trial relied on a finite 20-patient subset, the ordering of individual configurations may be affected by sampling variability. To avoid this influencing our conclusions, final classification performance was evaluated separately using patient-level stratified 5-fold cross-validation over the full cohort, with the selected augmentation re-applied at full resolution only within the training partition of each fold (Section 2.6).

The first stage was a grid search over the Cartesian product of five discrete levels per transformation parameter at three augmentation factors (0.5×, 1.0×, 1.5×). The second stage refined the best grid-search configuration via Bayesian optimization with Optuna (v4.7) [41], using the tree-structured Parzen estimator (TPE) sampler and median pruning for early stopping over continuous parameter intervals. The augmentation factor was searched over f∈{0.5,1.0,…,10.0}. Beyond 10×, augmented samples increasingly resemble one another and the quality scores plateau, yielding diminishing returns relative to the added computational and storage cost [16,17].

Each configuration was scored using three quality criteria, each scaled to [0, 100]. These criteria were deliberately separated from the final reported classification metrics (AUC, F1-score, and composite score) and from the feature-selection and classifier pipeline described in Section 2.6. Thus, augmentation parameters were not selected using the final evaluation metrics or the final selector–classifier pipeline. This reduced direct circularity between augmentation tuning and model evaluation. However, two criteria (robustness gain and prediction consistency) relied on class labels and a standalone Random Forest classifier, which was distinct from the final selector–classifier pipeline. We therefore interpret this preliminary optimization step as part of exploratory model design rather than as an independent validation procedure.

Distribution preservation assessed whether augmented features retained the statistical properties of the originals. For each radiomic feature, we compared the distributions of original and augmented samples using a two-sample Kolmogorov–Smirnov test and computed the median relative feature drift. The distribution score combined the proportion of features passing the KS test (60% weight) with the complement of the median drift (40% weight). No correction for multiple comparisons was applied, as the score was used for relative ranking rather than inferential testing.

Robustness gain measured whether adding augmented samples improved classification accuracy. Using a standalone Random Forest classifier with repeated stratified cross-validation, we defined the robustness score as a neutral-centered metric where no change in accuracy yielded a score of 50.

Prediction consistency quantified the proportion of augmented samples that received the same class label as their parent originals when classified by a model trained exclusively on original data.

#### 2.4.14. Selected Augmentation Factor

Optuna [41] was used in multi-objective mode to identify Pareto-optimal configurations for each augmentation strategy. The final configuration, including the expansion factor *f*, was selected from the Pareto front using the highest composite score. Across the 36 evaluated configurations, selected expansion factors ranged from f=0.5 to f=2.0, with strategy-specific values reported in Section 3.2.

#### 2.4.15. Shape-Descriptor Sensitivity of Spatial Augmentation

Because several discriminative features were shape- and diameter-related, we assessed whether spatial augmentations introduced substantial changes in lymph-node morphology. For each augmented sample, PyRadiomics shape descriptors were compared with those of the corresponding original lymph-node mask. Intensity-domain augmentations did not modify the segmentation masks and therefore left shape descriptors unchanged. Spatial transformations produced bounded morphology changes consistent with their predefined parameter ranges, without evidence of implausible nodal deformation. These checks were used to support the interpretation of augmentation effects on shape-derived radiomic features.

### 2.5. Radiomics Feature Extraction

Radiomics features were extracted from the original and augmented contrast-enhanced CT volumes using the corresponding lymph-node masks. Extraction was performed on the HU-preserved image representation after cropping, resampling, denoising, and HU clipping. The per-volume Z-score normalization used for the deep-learning comparison was not applied before PyRadiomics extraction; therefore, first-order features were interpreted as absolute HU-based descriptors. In total, 107 unfiltered PyRadiomics features were retained per lymph node, including first-order, shape, and texture descriptors [11,42].

### 2.6. Feature Selection and Classification

Given the high number of radiomic features relative to the cohort size, systematic feature selection was applied to derive compact models and reduce overfitting risk. We adapted the eliminative feature-selection framework from our prior work [21] for binary classification, evaluating five feature-selection approaches and seven classifiers, as summarized in Table 2. Candidate subsets ranged from 2 to 10 features in increments of 1 and from 20 to 90 features in increments of 10.

All classifiers used default scikit-learn hyperparameters without additional inner tuning, so that performance differences primarily reflected the effects of feature selection and augmentation rather than classifier-specific optimization. For ranking-based selectors, feature subsets consisted of the top *k* features by importance; for GA and RFE, subsets were directly optimized.

Optimal configurations were identified using Pareto front analysis, maximizing AUC, accuracy, and F1-score while minimizing the number of selected features. Pareto-optimal configurations were ranked by a composite score defined as the arithmetic mean of AUC, accuracy, and F1-score.

Performance was estimated using patient-level stratified five-fold cross-validation (StratifiedGroupKFold), ensuring that all samples from the same patient remained within the same fold. Within each fold, augmentation, feature scaling, feature selection, and model fitting were performed using only the training partition, and the learned transformations were applied to the held-out fold. Because model selection was not embedded within an independent outer validation loop, the reported results should be interpreted as exploratory model-selection estimates rather than unbiased estimates of external generalization performance.

Performance was assessed under five-fold cross-validation using three complementary metrics: AUC, accuracy, and F1-score. AUC was used as a threshold-independent measure of overall discrimination, whereas accuracy and F1-score were used to assess predictive performance at a fixed decision threshold. Accuracy and F1-score were computed using the default decision threshold of 0.5 applied to the predicted class-membership probabilities, following the same classification pipeline used in our prior work [21]. No clinically prespecified operating threshold was defined, and no threshold optimization was performed; therefore, sensitivity, specificity, positive predictive value, and negative predictive value at a defined clinical operating point were not reported. The three metrics were combined into a composite score, calculated as their arithmetic mean, to provide a pragmatic ranking criterion that captured both discrimination and fixed-threshold predictive performance. Equal weighting was used to avoid imposing an arbitrary preference among discrimination, classification accuracy, and precision–recall balance in the absence of a predefined clinical utility function. Accordingly, the composite score should be interpreted as a model-selection criterion rather than as a definitive measure of clinical utility, and alternative weighting schemes could alter the ranking of individual configurations.

## 3. Results

**Figure 3:**
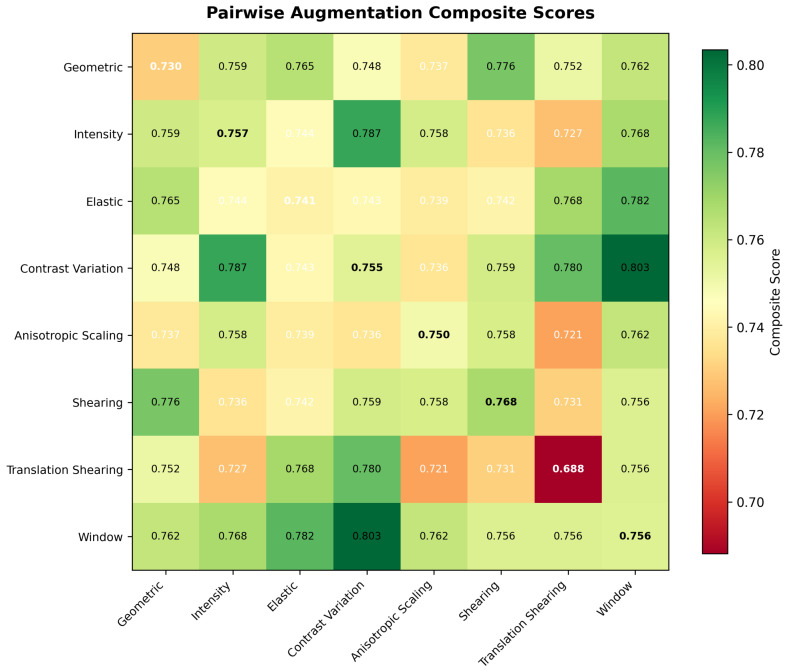
Pairwise augmentation composite score matrix. Diagonal entries correspond to single-strategy scores, and off-diagonal entries show pairwise combination scores. Grey cells indicate combinations that were not evaluated. Color scale ranges from red (lower) through yellow to green (higher).

The two-stage tuning protocol (grid search followed by Optuna optimization) produced final augmentation quality scores (the composite of distribution preservation, robustness gain, and prediction consistency defined in Section 2.4.13; range 0–100) for each strategy. Compared with grid search alone, Optuna refinement improved the quality score for three strategies (Geometric: +9.8, Anisotropic Scaling: +0.6, Contrast Variation: +0.1), left two unchanged (Shearing, Translation Shearing), and lowered it for three (Intensity: −8.1, Elastic: −5.0, Window: −1.9), with a mean absolute difference of 3.2 points. Despite slight degradation for some strategies, Optuna-refined parameters were retained for all to maintain a consistent optimization protocol; the final classification results were not materially affected, as confirmed by the sensitivity analysis below. Figure 3 provides an overview of the resulting composite scores across all single and pairwise augmentation configurations.

### 3.1. Impact of Feature Selection

Without feature selection, the best classification performance using all 107 radiomic features reached a mean composite score of 0.659 across five patient-level stratified cross-validation folds with an XGBoost classifier, corresponding to an AUC of 0.717, ACC of 0.631, and F1-score of 0.631. All metrics reported hereafter follow the same cross-validation scheme unless otherwise noted. Applying systematic feature selection improved performance across all metrics. The best configuration without augmentation used RFE with 20 features and a Random Forest classifier, achieving an AUC of 0.763, ACC of 0.740, and F1-score of 0.722 for a composite of 0.742, representing a 12.6% improvement over the no-selection baseline. This configuration served as the reference for all subsequent augmentation comparisons, as shown in Table 3.

### 3.2. Classification Performance Across Augmentation Strategies

Performance varied systematically across augmentation types. Table 4 summarizes each single-transform strategy’s best configuration, ordered by composite score. Among single strategies, Shearing achieved the highest composite of 0.768, followed by Intensity of 0.757 and Contrast Variation of 0.755. Geometric attained the highest individual AUC of 0.855 but a below-baseline composite of 0.730 due to lower ACC and F1-score. Two of five spatial strategies and both intensity strategies exceeded the feature-selection-only baseline composite. CT Window Variation was not carried into the ranked single-strategy comparison because its standalone feature drift was below 1%, although it contributed meaningfully in pairwise combinations. The complete ranking of all 36 single and pairwise augmentation configurations, with the no-augmentation baseline, is provided in Table 5.

The highest-ranked configuration overall was Window Contrast Variation, an intensity-plus-intensity pairing, using RF ranking with 40 features and an LDA classifier. It achieved a mean AUC of 0.831, ACC of 0.793, and F1-score of 0.787 for a composite of 0.803. Table 3 summarizes the top five configurations. All five were pairwise combinations, and four of five employed LDA as the classifier.

Window Contrast Variation showed gains across all metrics relative to baseline: AUC improved by 8.9%, ACC by 7.2%, and F1-score by 9.0%. Among selectors, RF ranking appeared most frequently in top configurations, displacing the baseline-optimal RFE. The optimal number of selected features ranged from 3 to 90, with a median of 40 across the top ten configurations. Notably, the fourth-ranked configuration (Contrast Variation Translation Shearing, RFE, Random Forest) achieved a mean AUC of 0.819 ± 0.042 with only three features, within 1.2 percentage points of the best 40-feature configuration. These three features were Maximum3DDiameter, Maximum2DDiameterColumn, and Maximum2DDiameterRow, the same shape descriptors that were independently selected by more than 75% of all strategies (Section 3.3).

### 3.3. Classification Performance and Feature Stability

The combined effect of feature selection and augmentation yielded a relative point-estimate improvement of 21.9% in composite score compared with the no-selection baseline, increasing from 0.659 to 0.803. This improvement occurred in two steps: feature selection increased the composite score from 0.659 to 0.742, corresponding to a 12.6% relative gain over the no-selection baseline, while augmentation further increased the composite score from 0.742 to 0.803, corresponding to an additional 8.2% relative gain over the feature-selection-only baseline. The augmentation-associated increase in composite score was accompanied by an AUC increase from 0.763 to 0.831, corresponding to an 8.9% relative point-estimate gain. Across the 36 augmentation configurations, most showed higher point-estimate AUC than the feature-selection-only baseline AUC of 0.763, and 26 of 36 showed higher point-estimate composite scores. The mean AUC across augmentation configurations was 0.810±0.023. However, individual augmentation-versus-baseline differences did not reach statistical significance.

**Figure 4:**
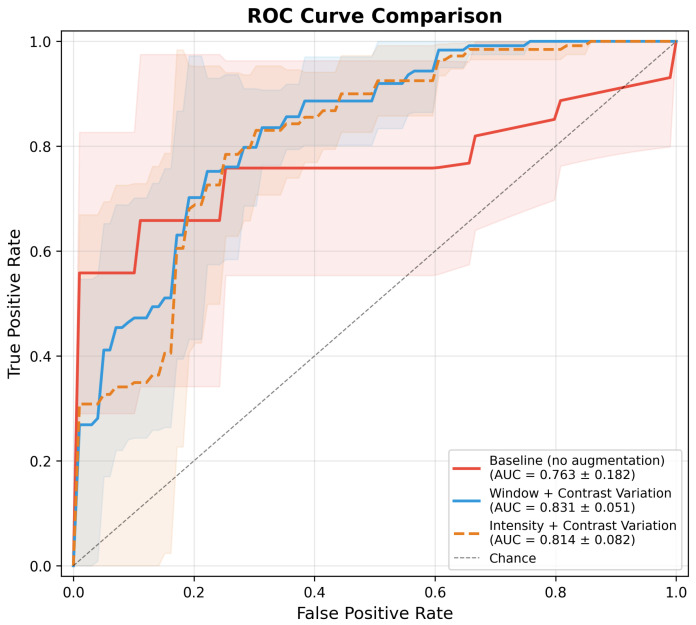
ROC curves for the baseline without augmentation, the best augmented configuration (Window Contrast Variation), and the second-best augmented configuration (Intensity Contrast Variation). Solid lines show the mean ROC curves across the five patient-level cross-validation folds. Each colored shaded band corresponds to the ROC curve of the same color and denotes ±1 standard deviation across folds, not a 95% confidence interval. Each held-out fold contained 11 patients with approximately balanced class distributions. Legend values indicate mean AUC ± standard deviation across folds.

Augmentation also reduced fold-to-fold variability: the baseline AUC standard deviation of 0.182 decreased to 0.051 for the best-performing configuration, a 3.6-fold reduction. Figure 4 presents mean ROC curves for the baseline and top two configurations.

Feature stability across augmentation strategies was limited, with a mean Jaccard similarity [43] of 0.241 between selected feature sets. Three shape descriptors were selected by more than 75% of strategies: Maximum3DDiameter (81%), Maximum2DDiameterColumn (78%), and Maximum2DDiameterRow (78%). An additional 14 features appeared in more than 50% of strategies.

To assess whether the frequent selection of size-related descriptors translated into sufficient standalone clinical performance, we performed an additional size-only baseline analysis on the original, non-augmented cohort using the same patient-level five-fold cross-validation framework. Maximum 3D diameter alone, used as a RECIST-like size proxy, achieved an AUC of 0.52. Logistic regression and Random Forest models using three diameter-based descriptors achieved AUCs of 0.45 and 0.68, respectively, while logistic regression using all four available diameter-based descriptors achieved an AUC of 0.61. In comparison, models using the full radiomics feature set of 107 features achieved AUCs of 0.77 with logistic regression and 0.74 with Random Forest. These findings indicate that size-related descriptors were frequently selected and contributed to the classification signal, but that size-only baselines performed below the full radiomics feature representation.

### 3.4. Cross-Scanner Sensitivity Analysis

To assess scanner vendor shift, we performed an exploratory leave-one-vendor-out sensitivity analysis using the manufacturer tags recorded in the CT DICOM headers. The cohort comprised 42 patients imaged on GE systems (21 vital, 21 non-vital) and 13 patients imaged on Siemens systems (6 vital, 7 non-vital). Models were trained on the GE partition and tested on the held-out Siemens partition. Feature scaling and feature selection were fitted on the GE training partition only and then applied unchanged to the Siemens test cases. Augmentation was applied only to the GE training partition, while the Siemens cases were evaluated as original, non-augmented cases.

For the evaluated Window Contrast Variation configuration with Linear Discriminant Analysis and 20 selected features, the test-set AUC on the 13 Siemens cases was 0.881 (95% CI 0.64–1.00). The corresponding unaugmented configuration evaluated on the same Siemens test set yielded an AUC of 0.786 (95% CI 0.48–1.00). Across evaluated selector–classifier combinations, AUC point estimates ranged from 0.30 to 0.88 on the Siemens test set. Because the Siemens test partition included only 13 patients, confidence intervals were wide, and the augmented estimate overlapped the unaugmented estimate. This analysis should therefore be interpreted only as an exploratory scanner-shift sensitivity check and does not establish scanner-level generalizability.

## 4. Discussion

This study systematically evaluated image-level data augmentation for radiomics-based classification of persistent cervical lymph node viability after chemoradiotherapy in HNSCC. Given the limited cohort of 55 patients relative to 107 extracted radiomic features, the findings should be interpreted as exploratory and require multi-center external validation before clinical application. The best augmented configuration, Window Contrast Variation, increased the composite score from 0.742 to 0.803, corresponding to an 8.2% relative augmentation-associated gain beyond the feature-selection-only baseline, following the 12.6% gain achieved by feature selection alone. This configuration also improved the point estimates of the individual performance metrics and reduced fold-to-fold AUC variability, with the AUC standard deviation decreasing from 0.182 to 0.051 across the five folds.

The composite score, defined as the arithmetic mean of AUC, accuracy, and F1-score, was used as a pragmatic ranking criterion rather than as a definitive clinical-performance measure. It was intended to capture both threshold-independent discrimination and fixed-threshold predictive performance, since AUC alone does not indicate how well a model performs at a given decision threshold. This distinction is illustrated by the Geometric strategy, which achieved the highest AUC among the evaluated configurations (0.855) but a lower composite score (0.730) because of reduced accuracy and F1-score. Thus, although this configuration ranked patients relatively well by predicted risk, its default decision threshold did not translate this ranking into equally strong class assignments. This divergence highlights the need for calibration assessment and clinically justified threshold selection before any clinical use.

In current clinical practice, determining whether a persistent cervical lymph node harbors residual viable tumor relies primarily on histopathological confirmation via salvage neck dissection or fine-needle aspiration cytology [24], or on serial clinical and imaging follow-up over at least 12 months [15]. Both approaches have limitations: surgery is invasive and may be unnecessary in patients with non-vital nodes, whereas prolonged observation can delay definitive management in patients with residual viable disease. However, contrast-enhanced CT alone cannot reliably distinguish post-treatment fibrosis from residual tumor [23], pointing to the need for additional quantitative imaging biomarkers.

Several findings support the clinical relevance of the extracted radiomic features. In one augmented model configuration, three diameter-based shape descriptors achieved relatively strong performance (AUC 0.819), consistent with the established role of nodal size as a clinical predictor [44]. However, the additional size-only baseline analysis performed on the original, non-augmented cohort (Section 3.3) showed that size alone was not sufficient to reproduce the performance of the full radiomics feature representation. Maximum 3D diameter alone achieved an AUC of 0.52, and size-only models using diameter-based descriptors achieved AUCs of 0.45 to 0.68, compared with AUCs of 0.74 to 0.77 for models using the full radiomics feature set. These findings suggest that lymph node size is important but incomplete, and that the full radiomics pipeline captures additional discriminatory information beyond simple diameter-based descriptors. This is clinically relevant because size-based assessment can lose specificity in the post-treatment setting, where reactive enlargement, necrosis, fibrosis, and treatment-related morphological changes may confound interpretation [23,24].

Intensity and contrast domain augmentations generally outperformed spatial transformations. This pattern is consistent with how radiomic features are computed: texture descriptors derived from grey-level co-occurrence, run-length, and size-zone matrices depend directly on intensity distributions [11]. Contrast-based perturbations alter the values from which these descriptors are calculated without substantially distorting spatial relationships, thereby encouraging classifiers to be robust to scanner- and protocol-dependent intensity variation. Spatial augmentations, by contrast, may introduce interpolation-related changes that affect texture stability. This observation suggests that augmentation design in radiomics pipelines should prioritize clinically plausible intensity and contrast variability while carefully controlling spatial transformations that may affect morphology or texture.

The two-stage parameter optimization framework, combining an initial grid search with Optuna refinement and three quality criteria: distribution preservation, robustness gain, and prediction consistency, addresses a recognized gap in which augmentation parameters are often selected manually [18]. The resulting best AUC of 0.831 lies in a similar range to the published performance of the Neck-Persistency-Net deep learning model (AUC 0.82 [15]) and to the CT-based lymph node response model reported by Zhang et al. [13], who achieved an AUC of 0.85 in a related induction chemotherapy setting in HNSCC. This comparison is contextual only: the models were not evaluated under identical data splits, preprocessing pipelines, or statistical testing, and no claim of equivalence is made. Nevertheless, the observation that a handcrafted radiomics pipeline performs in a similar range to a convolutional neural network on this task is notable, particularly given the lower computational cost and greater interpretability of radiomics models [8,9]. At the same time, the small cohort size limits the ability of both radiomics and deep learning approaches to establish robust generalizability.

Several limitations should be acknowledged. First, this was a single-center retrospective study including only 55 patients, which limits statistical power. The estimated minimum detectable AUC difference at 80% power was approximately 0.15 to 0.20, which is larger than the observed AUC gain between the feature-selection-only baseline and the best augmented configuration. Accordingly, although 26 of 36 configurations matched or exceeded the baseline composite score, this should be interpreted as a directional point-estimate trend rather than definitive evidence of benefit.

Second, all scans were acquired under a single institutional imaging protocol, although both GE and Siemens scanners were represented. The scanner vendor distribution was similar across vital and non-vital groups, but the Siemens subgroup was small. An exploratory GE-to-Siemens analysis was therefore informative only as a scanner-shift sensitivity check, not as definitive evidence of scanner-level generalizability. Scanner-level robustness must be confirmed in larger multi-vendor and multi-center cohorts with broader variation in scanner hardware, reconstruction settings, slice thickness, and contrast administration.

Third, the retrospective design limited full control over potential confounders, including the exact interval between treatment completion and restaging CT, acquisition-related parameters, and clinical variables that may influence post-treatment nodal appearance. Primary tumor site, p16 status, clinical T stage, and detailed treatment-regimen variables could not be reliably stratified by histopathological target-node status in the present analytic dataset and were therefore reported descriptively for the whole cohort rather than in the stratified baseline table. Although the available baseline and acquisition-related characteristics did not show obvious descriptive imbalance between vital and non-vital groups, larger datasets will be required to evaluate these factors more formally.

Fourth, although patient-level separation was maintained during cross-validation, augmentation parameter optimization was performed on cohort subsets before cross-validation rather than independently within each training fold. The optimization subsets were drawn from the same cohort used for subsequent model evaluation and therefore did not constitute independent hold-out cases. The separation between augmentation optimization and downstream evaluation was methodological rather than cohort-level, because augmentation parameters were selected using predefined quality criteria rather than the final cross-validated classification metrics. This reduces direct optimization of the final evaluation endpoints but does not eliminate the possibility of selection-related optimism. In addition, the extensive model-selection process across augmentation strategies, feature-selection methods, classifiers, and feature counts was not embedded within an independent outer validation loop. The selected pipeline should therefore be considered exploratory and requires confirmation either through nested validation or by testing prespecified augmentation settings in an independent external cohort.

Beyond addressing these limitations, several steps remain before the pipeline can be considered for clinical decision support. The Neck-Persistency-Net [15] and the radiomics pipeline capture complementary information, namely learned spatial representations and handcrafted quantitative descriptors. Combining both approaches in a late-fusion framework may improve performance, particularly in cases where size-based features are ambiguous. Integration of clinical variables such as tumor stage, treatment regimen, p16 status, and time to restaging may also provide context that imaging features alone cannot capture.

Most importantly, clinical utility must be assessed prospectively. Future studies should evaluate calibration, select clinically justified decision thresholds, and use decision-curve analysis to quantify the trade-off between false positives, which may lead to unnecessary salvage neck dissection, and false negatives, which may delay treatment of viable residual disease. The convergence of independent feature-selection algorithms on diameter-related shape descriptors suggests that lymph node morphology is an important driver of classification in this cohort. Whether this remains true in more heterogeneous multi-center data, where texture and intensity features may become more discriminative, remains an open question and motivates the validation studies outlined above.

## 5. Conclusions

This study suggests that optimized image-level augmentation may improve CT-based radiomics classification of persistent cervical lymph node viability in HNSCC. The best configuration, Window Contrast Variation, increased the point-estimate AUC from 0.763 to 0.831 over the feature-selection-only baseline, with gains in accuracy and F1-score that did not reach statistical significance. Shape descriptors measuring lymph node diameter were the most stable features across strategies, reinforcing their clinical relevance. Augmentation was also associated with lower fold-to-fold variability in this exploratory cohort. These findings warrant further investigation in larger clinical cohorts. Before clinical deployment, however, the pipeline requires external validation, prospective evaluation, calibration assessment, and decision-curve analysis to select a clinically justified threshold balancing unnecessary salvage neck dissection against missed viable disease.

## Figures and Tables

**Table 1:** Baseline acquisition and target-node characteristics stratified by histopathological nodal status. Age is reported as mean ± SD; categorical variables as counts; slice thickness and acquisition period as median [range]; and nodal-size descriptors as median [interquartile range]. Group differences are presented descriptively only.

Characteristic	Vital (*n* = 27)	Non-Vital (*n* = 28)	Overall (*n* = 55)
Age, years (mean ± SD)	60.2 ± 12.0	62.3 ± 8.3	61.3 ± 10.2
Sex, *n* (male/female)	24/3	23/5	47/8
Scanner vendor, *n* (GE/Siemens)	21/6	21/7	42/13
Slice thickness, mm (median [range])	2.50 [0.625–3.75]	2.50 [0.625–3.75]	2.50 [0.625–3.75]
Acquisition period, year (median [range])	2015 [2008–2021]	2016 [2010–2020]	2015 [2008–2021]
Maximum 3D diameter, mm (median [IQR])	29.0 [24.1–37.0]	27.3 [19.0–42.2]	28.2 [21.3–37.7]
Minor-axis length, mm (short-axis proxy; median [IQR])	14.0 [11.3–16.4]	12.4 [9.6–14.9]	12.9 [10.0–15.8]

**Table 2:** Feature-selection and classification methods and their key configuration settings, adapted from [21]. BACC, balanced accuracy.

Method	Configuration
**Feature Selectors**	
Genetic Algorithm (GA)	Population = 5000; generations = 500; two-point crossover ($p_{\mathrm{cx}}=0.5$); bit-flip mutation ($p_{\mathrm{mut}}=0.2$, $p_{\mathrm{ind}}=0.05$); tournament selection (k=3); early stopping after 50 stagnant generations; fitness = BACC using inner 2-fold CV with LDA, penalized by 0.05×|selected−target|
Random Forest (RF)	Gini importance; $n_{\mathrm{estimators}}=100$
XGBoost	Split-gain importance; $n_{\mathrm{estimators}}=100$; L1/L2 regularization
Sparse Discriminant Analysis (SDA)	Elastic-net-penalized discriminant analysis using the R package sparseLDA; tolerance = $10^{-6}$; stop = $-n_{\mathrm{target}}$
Recursive Feature Elimination (RFE)	Backward elimination with an LDA estimator; step size = 1
**Classifiers**	
Linear Discriminant Analysis (LDA)	Default settings
Logistic Regression	L2 penalty
Support Vector Machine (SVM)-Linear	C=1.0
SVM-RBF	C=1.0; γ=scale
SVM-Sigmoid	C=1.0; γ=scale; coef0=0
Random Forest (RF)	$n_{\mathrm{estimators}}=100$
XGBoost	$n_{\mathrm{estimators}}=100$; L1/L2 regularization

**Table 3:** Top five augmentation configurations ranked by composite score among 37 evaluated configurations (36 augmented configurations and the baseline). The unaugmented baseline and the single strategy with the highest AUC are shown for reference. All metrics are reported as mean ± SD across five cross-validation folds.

Augmentation	Selector	No. Features	Classifier	AUC	ACC	F1	Score
**Top five by composite score**							
Window + Contrast Var.	RF	40	LDA	0.831 ± 0.051	0.793 ± 0.044	0.787 ± 0.069	0.803
Intensity + Contrast Var.	XGBoost	50	LDA	0.814 ± 0.082	0.781 ± 0.089	0.768 ± 0.105	0.787
Elastic + Window	RF	40	LDA	0.837 ± 0.101	0.755 ± 0.109	0.755 ± 0.101	0.782
Contrast Var. + Trans. Shear.	RFE	3	RF	0.819 ± 0.042	0.763 ± 0.073	0.758 ± 0.036	0.780
Geometric + Shearing	GA	50	LDA	0.822 ± 0.065	0.759 ± 0.070	0.748 ± 0.089	0.776
**Reference configurations**							
Baseline (No Augmentation)	RFE	20	RF	0.763 ± 0.182	0.740 ± 0.135	0.722 ± 0.161	0.742
Geometric (highest AUC)	RFE	60	SVM-Linear	0.855 ± 0.155	0.703 ± 0.206	0.632 ± 0.213	0.730

**Table 4:** Best-performing configuration for each single-transform augmentation strategy, ordered by composite score. Each row shows the selector, classifier, and feature-count combination that achieved the highest composite score for that strategy; the best AUC for a given strategy may occur at a different selector–classifier combination. The no-augmentation baseline is included for reference. All metrics are means ± SD across five cross-validation folds.

Strategy	Selector	No. Features	Classifier	AUC	ACC	F1	Score
Shearing	GA	70	XGBoost	0.820 ± 0.159	0.751 ± 0.163	0.734 ± 0.159	0.768
Intensity	SDA	50	LDA	0.805 ± 0.099	0.748 ± 0.063	0.718 ± 0.057	0.757
Contrast Variation	GA	10	LDA	0.781 ± 0.067	0.752 ± 0.045	0.732 ± 0.046	0.755
Anisotropic Scaling	GA	80	XGBoost	0.828 ± 0.162	0.730 ± 0.167	0.691 ± 0.234	0.750
Original (No Aug)	RFE	20	RF	0.763 ± 0.182	0.740 ± 0.135	0.722 ± 0.161	0.742
Elastic	RFE	8	RF	0.757 ± 0.083	0.742 ± 0.071	0.724 ± 0.086	0.741
Geometric	RFE	60	SVM-Linear	0.855 ± 0.155	0.703 ± 0.206	0.632 ± 0.213	0.730
Translation Shearing	GA	70	XGBoost	0.809 ± 0.113	0.659 ± 0.143	0.597 ± 0.180	0.688

**Table 5:** Full ranking of all 36 augmentation configurations by composite score, with the no-augmentation baseline for reference. ^†^, below baseline composite; ^‡^, tied with baseline.

Strategy	Selector	No. Features	Classifier	AUC	ACC	F1	Score
Window + Contrast Variation	RF	40	LDA	0.831 ± 0.051	0.793 ± 0.044	0.787 ± 0.069	0.803
Intensity + Contrast Variation	XGBoost	50	LDA	0.814 ± 0.082	0.781 ± 0.089	0.768 ± 0.105	0.787
Elastic + Window	RF	40	LDA	0.837 ± 0.101	0.755 ± 0.109	0.755 ± 0.101	0.782
Contrast Variation + Translation Shearing	RFE	3	RF	0.819 ± 0.042	0.763 ± 0.073	0.758 ± 0.036	0.780
Geometric + Shearing	GA	50	LDA	0.822 ± 0.065	0.759 ± 0.070	0.748 ± 0.089	0.776
Elastic + Translation Shearing	RFE	3	RF	0.814 ± 0.046	0.740 ± 0.070	0.751 ± 0.041	0.768
Shearing	GA	70	XGBoost	0.820 ± 0.159	0.751 ± 0.163	0.734 ± 0.159	0.768
Intensity + Window	RF	40	LDA	0.828 ± 0.025	0.742 ± 0.055	0.733 ± 0.072	0.768
Geometric + Elastic	RFE	40	LDA	0.799 ± 0.057	0.752 ± 0.072	0.744 ± 0.099	0.765
Geometric + Window	RFE	40	LDA	0.810 ± 0.084	0.743 ± 0.088	0.733 ± 0.117	0.762
Window + Anisotropic Scaling	RFE	90	LDA	0.790 ± 0.028	0.747 ± 0.035	0.749 ± 0.049	0.762
Geometric + Intensity	RFE	50	LDA	0.776 ± 0.029	0.749 ± 0.028	0.753 ± 0.012	0.759
Shearing + Contrast Variation	RFE	3	RF	0.808 ± 0.053	0.727 ± 0.081	0.742 ± 0.052	0.759
Intensity + Anisotropic Scaling	RFE	70	XGBoost	0.843 ± 0.109	0.721 ± 0.087	0.711 ± 0.106	0.758
Shearing + Anisotropic Scaling	GA	70	LDA	0.810 ± 0.105	0.748 ± 0.133	0.715 ± 0.160	0.758
Intensity	SDA	50	LDA	0.805 ± 0.099	0.748 ± 0.063	0.718 ± 0.057	0.757
Window + Translation Shearing	RFE	30	LDA	0.827 ± 0.115	0.735 ± 0.146	0.706 ± 0.206	0.756
Window + Shearing	RF	40	LDA	0.792 ± 0.080	0.738 ± 0.057	0.739 ± 0.069	0.756
Window	RF	60	LDA	0.767 ± 0.076	0.759 ± 0.105	0.740 ± 0.138	0.756
Contrast Variation	GA	10	LDA	0.781 ± 0.067	0.752 ± 0.045	0.732 ± 0.046	0.755
Geometric + Translation Shearing	RFE	40	LDA	0.794 ± 0.078	0.735 ± 0.079	0.725 ± 0.098	0.752
Anisotropic Scaling	GA	80	XGBoost	0.828 ± 0.162	0.730 ± 0.167	0.691 ± 0.234	0.750
Geometric + Contrast Variation	RFE	60	XGBoost	0.744 ± 0.151	0.756 ± 0.096	0.742 ± 0.108	0.748
Intensity + Elastic	XGBoost	30	RF	0.788 ± 0.132	0.729 ± 0.084	0.716 ± 0.111	0.744
Elastic + Contrast Variation	GA	60	XGBoost	0.777 ± 0.158	0.734 ± 0.121	0.719 ± 0.142	0.743
Elastic + Shearing ^‡^	RFE	5	RF	0.771 ± 0.036	0.731 ± 0.091	0.723 ± 0.083	0.742
Original (No Aug)	RFE	20	RF	0.763 ± 0.182	0.740 ± 0.135	0.722 ± 0.161	0.742
Elastic ^†^	RFE	8	RF	0.757 ± 0.083	0.742 ± 0.071	0.724 ± 0.086	0.741
Elastic + Anisotropic Scaling ^†^	RFE	20	XGBoost	0.750 ± 0.084	0.725 ± 0.069	0.741 ± 0.072	0.739
Geometric + Anisotropic Scaling ^†^	GA	40	RF	0.766 ± 0.126	0.732 ± 0.075	0.713 ± 0.102	0.737
Contrast Variation + Anisotropic Scaling ^†^	XGBoost	60	LDA	0.787 ± 0.136	0.718 ± 0.172	0.705 ± 0.194	0.736
Intensity + Shearing ^†^	RFE	3	RF	0.760 ± 0.088	0.723 ± 0.094	0.725 ± 0.085	0.736
Shearing + Translation Shearing ^†^	RFE	3	RF	0.773 ± 0.087	0.705 ± 0.081	0.714 ± 0.064	0.731
Geometric ^†^	RFE	60	SVM-Linear	0.855 ± 0.155	0.703 ± 0.206	0.632 ± 0.213	0.730
Intensity + Translation Shearing ^†^	GA	70	SVM-Sigmoid	0.765 ± 0.131	0.717 ± 0.074	0.700 ± 0.105	0.727
Anisotropic Scaling + Translation Shearing ^†^	XGBoost	9	LR	0.760 ± 0.191	0.706 ± 0.118	0.697 ± 0.157	0.721
Translation Shearing ^†^	GA	70	XGBoost	0.809 ± 0.113	0.659 ± 0.143	0.597 ± 0.180	0.688

## Data Availability

The data presented in this study are available on request from the corresponding author.
